# Exploring Fast Fingerprint Construction Algorithm for Unmodulated Visible Light Indoor Localization

**DOI:** 10.3390/s20247245

**Published:** 2020-12-17

**Authors:** Chenqi Shi, Xinyv Niu, Tao Li, Sen Li, Chanjuan Huang, Qiang Niu

**Affiliations:** 1 School of Computer Science and Technology, China University of Mining and Technology, Xuzhou 221116, China; shichenqi@cumt.edu.cn (C.S.); 06171904@cumt.edu.cn (X.N.); li_t@cumt.edu.cn (T.L.); 08182754@cumt.edu.cn (S.L.); 14184450@cumt.edu.cn (C.H.); 2 China Mine Digitization Engineering Research Center, Ministry of Education, Xuzhou 221116, China

**Keywords:** visible light indoor position, fingerprint location, image process

## Abstract

The study of visible light indoor position has received considerable attention. The visible light indoor position has problems such as deployment difficulty and high cost. In our system, we propose a new fingerprint construction algorithm to simplify visible light indoor position. This method can realize the rapid construction of a visible fingerprint database and prove that the fingerprint database can be used repeatedly in different environments. We proved the theoretical feasibility of this method through theoretical derivation. We carried out extensive experiments in two classic real indoor environments. Experimental results show that reverse fingerprinting can be achieved. In 95% of cases, the positioning accuracy can be guaranteed to be less than 10 cm.

## 1. Introduction

The rapid development of the Internet of Things (IoTs) promotes the development of smart devices [1,2,3,4]. Indoor positioning greatly expands the capabilities of these devices and therefore attracts a great amount of research work. The global positioning system has limitations in indoor positioning [5,6,7,8], and researchers have proposed the use of Wireless Fidelity (Wi-Fi), ultra-wideband (UWB), ultrasonic wave, and visible light positioning (VLP) technologies [9,10,11,12,13]. However, the main problem with Wi-Fi signals is the multipath effect. Although both UWB and ultrasonic wave can provide submeter positioning accuracy (0.1 m), the high cost to deploy these systems is their main disadvantage [14,15,16,17,18,19].

Most visible light indoor positioning needs the support of visible light communication (VLC) technology [20,21,22,23,24]. VLC requires additional equipment to adjust the frequency of light, and most of them require photodiodes as the receiving end, which increases deployment costs [25,26,27,28]. Some scholars recently proposed visible light positioning (VLP) methods which do not need VLC support to match the visible light fingerprint dataset [9,29,30,31,32]. However, because the fingerprint database is affected by the environment, it takes a great amount of work to collect fingerprints frequently [33,34,35,36,37]. Therefore, the rapid establishment of fingerprint database has become the focus of this work. Fingerprint recognition is the main method of indoor visible light positioning [38,39,40,41,42,43]. Its disadvantage is that the process of fingerprint collection is cumbersome and high-cost for changing visible light fingerprints. Therefore, we proposed a simple fingerprint acquisition method to realize the low-cost VLP [44,45,46,47,48,49,50].

To address these issues, we explored a new fingerprint collection method and called it the reverse fingerprint collection method. Furthermore, we use this method for visible light indoor positioning, which does not need VLC support. Using ambient light or a headlamp, it can complete the position of a person (carrying a mobile phone that can shoot) and smart devices (with a camera). We study the principle of light reflection and the principle of light propagation in a straight line and derive a method to establish a fingerprint database in the entire indoor space quickly. This method lays a foundation for simplifying the fingerprint collection process. First, using the principle of color polarization of light, different interference patterns are obtained through polarizers and birefringent crystals. Then, we use the principle of straight-line propagation of light to quickly, and with low cost, construct the visible light positioning fingerprint of the entire space.

In the actual design of the reverse fingerprint method, we only need to use commercial white light or sunlight as the light source. We use low-priced polarizers and ordinary transparent tape with birefringence characteristics to make color polarizers. Each color polarizer costs no more than 10 cents. The color polarizing plate is attached in front of the light source; the interference pattern is projected on the white reflective plate. The interference pattern on the reflector is photographed and recorded, and a full-space positioning fingerprint library is obtained after image processing.

The specific objective of this study was to improve the unmodulated visible light position method [9]. This paper proposes a novel fingerprint collection method, which significantly simplifies the difficulty and workload of fingerprint collection in the visible light position system, and experimentally obtains how different relative positions of positioning originals will affect the positioning results.

The contributions of our work mainly include the following aspects:

- We proposed a new reverse fingerprint collection method to realize the rapid collection and deployment of fingerprint information with the change of environment. In addition, through theory and practice, we prove the reusability of reverse-collected fingerprints in different environments;
- We combine the theory of light propagation and image processing method for the first time, which significantly reduces the workload and time cost of constructing a visible light location fingerprint database. Meanwhile, the fingerprint library of the whole indoor environment can be obtained in a short time;
- We conducted multiple sets of experiments and compared the impact of fingerprints collected in different environments on the positioning results. The reverse fingerprinting can be achieved. In 95% of cases, the positioning accuracy can be guaranteed to be less than 10 cm.

The organization of the remainder is as follows. Section 2 introduces related work. Section 3 presents position basics of our work. Section 4 presents the localization model of the reverse fingerprint collection method. Section 5 introduces experiment of the reverse fingerprint collection method. Section 6 presents the evaluation results of the reverse fingerprint collection method. Section 7 concludes this work.

## 2. Related Work

Most VLPs use VLC technology as support. Some of the representative works are as follows. In [4], the authors maximized the communication speed of VLC while implementing VLP and lighting functions. A VLP system based on VLC and a genetic algorithm was proposed by the authors of [12]. In [15], the authors combined the radio frequency signal and visible light signal to build a portable and movable hybrid measuring element and use it for underground positioning. The authors of [5] expanded the number of LEDs used for communication and positioning to four, and added a four-quadrant algorithm as an auxiliary receiving scheme. The authors of [17] improved the difference algorithm to be suitable for visible light signals and subsequently used it for positioning research. Due to the complex hardware changes of these algorithms, they are limited in daily applications. Therefore, it is necessary to explore visible light positioning methods that do not require hardware changes.

In [7], the authors combined machine learning with visible light and USES Angle of arrival for indoor targeting, and they ended up using quadrant solar cells as a way to increase progress. The literature [1] used improved reception devices, programmable beacons, and embedded positioning programs in smartphones to achieve simple and low-cost visible light indoor positioning. The authors of [6] proposed a grid sampling scheme in the location domain. In [21], they used the determinant of the cost function to derive a more accurate and accurate visible light indoor algorithm; however, no environmental comparison was made. In [8], the visible light indoor positioning system and its algorithm were systematically analyzed, and suggestions for future research directions were put forward, providing inspiration for our work. The authors of [25] improved the scheme of using smartphones as visible signal receivers, and based on this, a new VLP solution was proposed. The authors of [9] showed RainbowLight, a novel method of using optical principles and modifying them for indoor positioning. These methods mainly inspire us to explore the reverse fingerprint position method.

## 3. Position Basics

**Figure 1:**
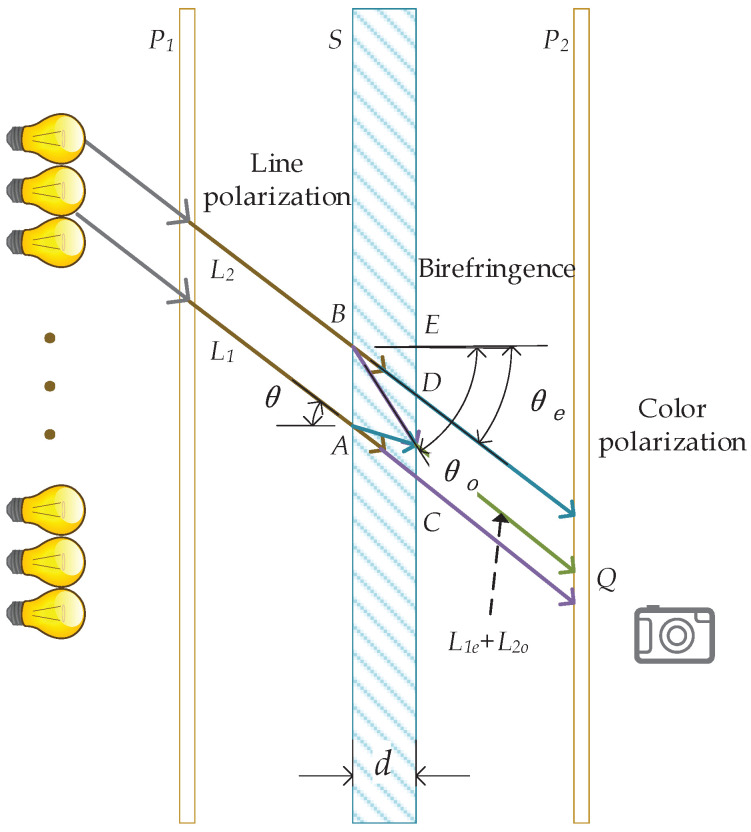
Illustration of optical ray. The birefringent plate *S* is placed between the polarizing plate $P_{1}$ and $P_{2}$. As light passes through, this group of units produces a color polarization effect.

In this section, we show the basic principles of the position for our method. The position method uses the phenomenon of color polarization of light. When light passes through the color polarizer, it will present different colors in different positions. The phenomenon of color polarization consists of polarization and birefringence. The light path of color polarization is shown in Figure 1.

### 3.1. Polarization

A light wave is a transverse wave. Polarization is a phenomenon that occurs only with transverse waves (also the most obvious sign that they are different from longitudinal waves). Natural light and LED light in each polarization direction are random and irregular. As the light passes through the polarizer, the electric vector is hindered by the spatial distribution, thereby losing the symmetry of the light wave vibration direction. Therefore, in the process of light propagation, only one kind of vibration is included, and its vibration direction is always kept in the same plane. This kind of light is called linear polarization light. As shown in Figure 1, natural light passes through the polarizing plate *P* and becomes linearly polarized light having an amplitude *A*. The polarization direction of the polarized light is parallel to the transmission axis of the polarizer.

### 3.2. Birefringence

When light enters a birefringent crystal, to get an incident along the direction of the crystal’s optical axis, the speed of light propagation will also change due to the direction of light vibration. This phenomenon is known as birefringence. The incident light will be broken into two rays whose vibration directions are perpendicular to each other. The two rays have different propagation speeds and have different refractive indices. One follows the general law of refraction, called the ordinary ray (o-ray), between the two kinds of refracted light produced by birefringent crystals. The other does not follow the ordinary law of refraction and is called the extraordinary ray (e-ray). In crystals, the propagation speed of the o-ray is independent of the propagation direction and is constant. The propagation speed of an e-ray is a variable related to the direction of propagation. There is a particular direction inside the crystal, and the speed of the e-ray traveling in this direction is equal to the speed of the o-ray. Due to its anisotropic properties, plastic film has a phenomenon of birefringence when light passes through. They can be used in experiments to replace birefringent crystals.

### 3.3. Color Polarization

The two beams have the same frequency and the same polarization direction, and have a fixed phase difference, which fully meets the interference conditions, so interference will occur. As shown in Figure 1, when they are projected on the polarizer $P_{2}$, two situations will occur: (1) $L_{e}$ and $L_{o}$ are projected to the same direction of $P_{2}$. At this time, $L_{e_{2}}$ and $L_{o_{2}}$ interfere without any additional phase difference. (2) $L_{e}$ and $L_{o}$ are projected to the two directions of $P_{2}$. The interference of $L_{e_{2}}$ and $L_{o_{2}}$ will produce an additional phase difference of π. Thus, linear polarized incident after polaroid and birefringence $L_{e_{2}}$ and $L_{o_{2}}$ phase caused by the part of the $\delta_{1}$ and $\delta_{2}$. $\delta_{1}$ is generated by the thickness as the birefringent crystal *d*, i.e., $\delta_{1}$ is the reason for different colors. $\delta_{2}$ with $L_{e}$ and $L_{o}$ projection onto the $P_{2}$, if two lights are in the same direction, $\delta_{2}$ = 0, otherwise $\delta_{2}$ = π.

## 4. Reverse Fingerprint Position Method

**Figure 2:**
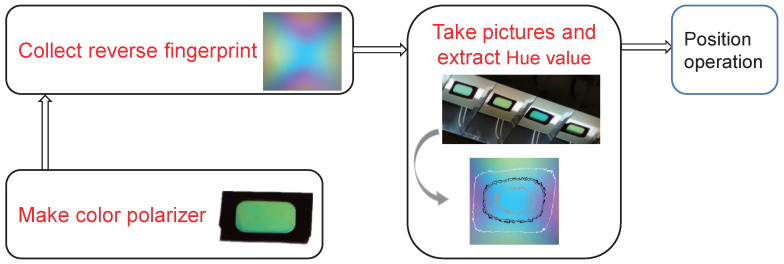
Reverse fingerprint location architecture.

In this section, we will first introduce the system principle of the reverse fingerprint collection method. Then, we will prove that, using this method, the fingerprint collected in one situation can be used for positioning in many different environments. The structural logic of the reverse fingerprint localization method is shown in Figure 2.

### 4.1. Reverse Fingerprint

As described in Section 3, after passing through the color polarizer, the wavelength value of the light will change, so the light will also have a different color. We use the principle of light reflection. When illuminating an object, the color of the object is determined by the light reflected or transmitted by the object, and the white opaque object reflects all colors of light. Therefore, if we put the color polarizer in front of the light source and place a white partition in front of the color polarizer, the white partition will reflect a unique polarization pattern. Since the light rays propagate in a straight line and the light path is reversible, taking any point on the white partition, the color of the light reflected at this point is the same as the color seen at the point where the color polarizer is taken. Therefore, the reflection pattern on the white partition can be photographed as the fingerprint pattern of the color polarizer. The method of using this pattern as fingerprint data for positioning is the reverse fingerprint positioning method.

Then, we place film in the polarization pattern and extract the fingerprint from it. We added some marker points and marker lines on the whiteboard. The function of the marker points is to record the projection point at the center of the color polarizer, while the function of the marker lines is to record the direction of the color polarizer. In this paper, the fingerprint picture cut into a square shape. The detailed steps of the method are shown in Figure 2.

We theoretically proved that under the condition of using the same type of color polarizer, fingerprints collected in one environment could be used in many different environments.

After the polarized light is incident on the surface of the birefringent crystal, intuitively, a beam of light is divided into two beams, ordinary light and extraordinary light. When entering the crystal, the phase difference between the two beams of light is zero, after passing through the wave chip with a thickness of *d*. The phase difference between e-light and o-light is $\delta_{1}$, where $n_{o}$ is the refractive of ordinary light and $n_{e}$ is the refractive of extraordinary light.

(1) $$\delta_{1}=\frac{2\pi }{\lambda }\left(n_{o}-n_{e}\right)d.$$

O-ray and e-ray pass through the birefringent crystal and enter another polarizer, and only the partial amplitudes $L_{e_{2}}$ and $L_{o_{2}}$ along the direction of the transmission axis can pass through $P_{2}$. As shown in Figure 1, respectively:

(2) $$\begin{array}{c} A_{e_{2}}=A_{1}\cos\left(\theta_{1}-\theta_{2}\right),\\ A_{o_{2}}=A_{2}\sin\left(\theta_{1}-\theta_{2}\right). \end{array}$$

### 4.2. Position

**Figure 3:**
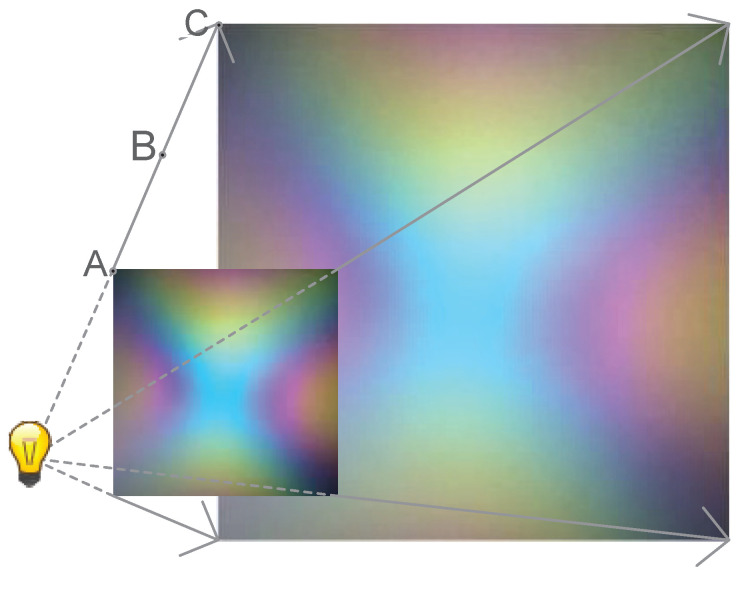
Illustration of fingerprint mapping.

First, the fingerprint image is sampled to improve the locating speed without affecting the locating accuracy. According to the straight-line propagation of light, the fingerprint pattern is expanded to the whole space. As shown in Figure 3, the color of the color polarizer observed at points B and C should be the same as that of Point A. Thus, the hue fingerprint has an equal value at both points, and similarly, on a ray with a light source as the vertex, the hue fingerprint has the same amount. Using the theory of straight-line propagation of light, mapping the color fingerprint collected at a fixed distance to any distance, the color fingerprint of the whole space can be obtained.

As shown in Figure 1, we use θ to indicate the incident angle of $L_{1}$ and $L_{2}$ to *S*, and thickness of *S* is *d*. $\theta_{o}$ and $\theta_{e}$ are refraction angle of ordinary ray and extraordinary ray of $L_{1e}$ and $L_{2o}$, respectively. γ is the angle between optic axis and the projection of incident light. $n_{air}$ is the refractive index of light in air, $n_{o}$ and $n_{e}$ are the refractive indexes of ordinary light and extraordinary light in birefringent crystals, respectively. The optical path different of $L_{1e}$ and $L_{2o}$ at point *Q* is $\delta_{1}$, which can be calculated as:

(3) $$\delta_{1}=\bar{FA}n_{air}+\bar{AD}n_{e}-\bar{BD}n_{o}=d\left(\tan\theta_{o}-\tan\theta_{e}\right)\sin\theta n_{\mathrm{air}}+\frac{d}{\cos\theta_{e}}n_{e}-\frac{d}{\cos\theta_{o}}n_{o}.$$

In Equation (3), $\bar{FA}$, $\bar{AD}$ and $\bar{BD}$ are the actual distances between two points from *A* to *F*, from *A* to *D*, and from *B* to *D*. So, we have:

(4) $$\delta_{1}=d\left(n_{e}\cos\theta_{e}-n_{o}\cos\theta_{o}\right).$$

Inspired by [9], in birefringence, the direction vector of the optical axis is $e_{a}$; the direction vector of ordinary rays is $e_{ko}$, and the direction vector of special rays is $e_{ke}$, as shown below:

(5) $$e_{a}=\left(\cos\gamma ,\sin\gamma ,0\right),$$

(6) $$e_{ko}=\left(\sin\theta_{o},0,\cos\theta_{o}\right),$$

(7) $$e_{ke}=\left(\sin\theta_{e},0,\cos\theta_{e}\right).$$

The angle between $e_{a}$ and $e_{ke}$ can be written as α. According to Equations (5) and (7), we have:

(8) $$\cos\alpha =e_{a}\cdot e_{ke}=\cos\gamma \sin\theta_{e}.$$

In birefringent materials, the refractive index of unusual light is not fixed and will change with the change of incident angle. $n_{e}$ is the refractive index of extraordinary ray in birefringent material; thus, we know that:

(9) $$n_{e}=\frac{N_{o}N_{e}}{\sqrt{N_{o}^{2}\sin^{2}\alpha +N_{e}^{2}\cos^{2}\alpha }}=\frac{N_{o}N_{e}}{\sqrt{N_{o}^{2}+\left(N_{e}^{2}-N_{o}^{2}\right)\cos^{2}\alpha }}.$$

For a fixed birefringent material, the main refractive index is a fixed value. According to Equations (8) and (9), we have:

(10) $$n_{e}=\frac{N_{o}N_{e}}{\sqrt{N_{o}^{2}+\left(N_{e}^{2}-N_{o}^{2}\right)\cos^{2}\gamma \sin^{2}\theta_{e}}}.$$

$n_{o}$ is the refractive index of ordinary ray in birefringent material. According to Snell’s Law, the following formula can be derived:

(11) $$n_{air}\sin\theta =n_{e}\sin\theta_{e}=n_{o}\sin\theta_{o}.$$

where $n_{air}$ is similar to one, which is the refractive index in air. Thus, we know that:

(12) $$n_{e}=\frac{\sin\theta }{\sin\theta_{e}}.$$

According to Equations (9) and (12), we have:

(13) $$\theta_{e}=\arcsin\sqrt{\frac{\sin^{2}\theta }{N_{e}^{2}-\sin^{2}\theta \left(\frac{N_{e}^{2}}{N_{o}^{2}}\cos^{2}\gamma -\cos^{2}\gamma \right)}}.$$

Finally, according to Equations (12) and (13), we have:

(14) $$n_{e}=\sqrt{N_{e}^{2}-\sin^{2}\theta \left(\frac{N_{e}^{2}}{N_{o}^{2}}\cos^{2}\gamma -\cos^{2}\gamma \right)}.$$

Because the optical path difference is:

(15) $$\delta_{1}=d\left(n_{e}\cos\theta_{e}-n_{o}\cos\theta_{o}\right).$$

We substitute $n_{e}$ , $\theta_{e}$ , and $n_{o}$ , $\theta_{o}$ into Equation (15):

(16) $$\delta_{1}=d\left(\sqrt{N_{e}^{2}-\sin^{2}\theta \left(\sin^{2}Y+\frac{N_{e}^{2}}{N_{o}^{2}}\cos^{2}\gamma \right)}-\sqrt{N_{o}^{2}-\sin^{2}\theta }\right).$$

$N_{e}$ and $N_{e}$ are refractive indices of *S*. The index of refraction is a fixed value relative to a fixed material. γ is the angle between the projection of incident light onto the plane of incidence and the optical axis of the plane of incidence.

For a specific wavelength of light λ, the phase different $\delta_{2}$ at point *D* in Figure 1 can be described as:

(17) $$\delta_{2}=\delta_{1}\frac{2\pi }{\lambda }.$$

As can be seen from the above, $\delta_{1}$ is the reason for different colors. It can be seen that the right part of Equation (14) is only related to angle and distance value and is unrelated to the incident light and ambient light conditions. This means that the color of the color polarizer has no connection with the ambient light, only connected with the collection location. Therefore, as long as the color information of the color polarizing plate is obtained, the shooting position information can be inferred from the fingerprint pattern. Because hue is the primary characteristic of color, it is the most accurate standard for distinguishing different colors. Here, we choose the hue value as an indicator to identify different colors.

**Figure 4:**
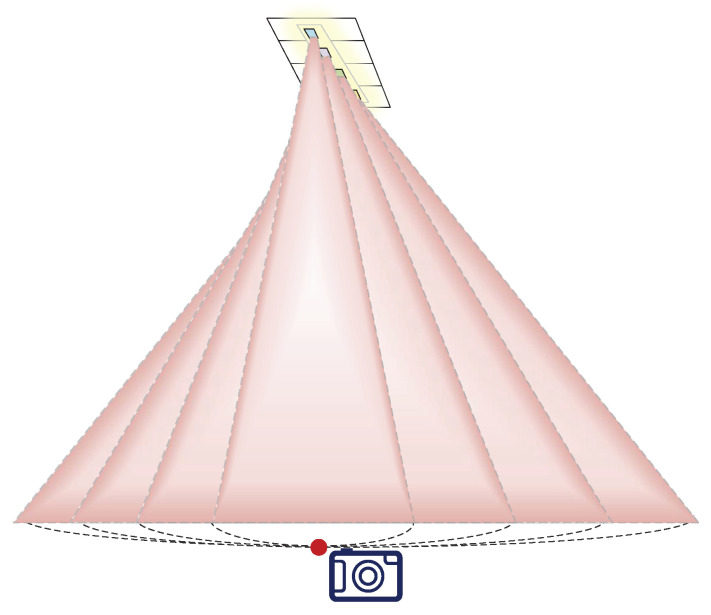
Illustration of location.

During positioning, the camera is only needed to point at the light source to shoot, and extract the color value of the color polarizer on the light source. Then, measure the angle between the light source where the color polarizer is located and the shooting device, a set of rays approximately distributed on the surface of the cone is made, and multiple (four in this paper) sets of rays are measured, and the intersection of these rays is found. The intersection location is the location of the shooting device, as shown in Figure 4.

## 5. Experiment

**Figure 5:**
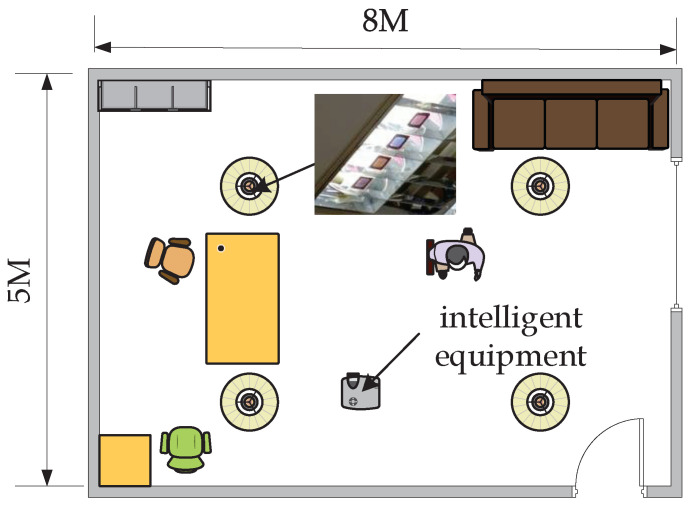
Illustration of location.

The experiment can be divided into two parts. The first part is to make color polarizers and to collect reverse fingerprints. The second part is to take pictures and position them. The time cost of calculating a set of positions is about 1–30 s. The experimental scene is shown in Figure 5.

### 5.1. Collect Reverse Fingerprint

As shown in Figure 1, we first need to make a color polarizer. We use a polarizer with a polarization angle of 90 degrees as the polarizing crystal and a transparent tape as the birefringent crystal to make color polarizers. Attach the birefringent crystal between the two polarizers. Note that the polarization directions of the two polarizers cannot be parallel. We rotated one of them by 90 degrees. The cost of a color polarizer is meager, no more than 5 cents each.

Since the hue fingerprint corresponds to the angle value of the shooting point and the polarizing color plate, the position of the shooting point can be determined using multiple color polarizers. Therefore, the first part is to establish a fingerprint database. This part is to establish the mapping between tone value and direction value, as shown in Figure 3. For polarizers of the same color, it is only necessary to create a fingerprint map once—that is, to establish a fingerprint database. The second part is to coordinate positioning. In this part, the camera uses a color polarizer with multiple different positions and different rotation angles, as shown in Figure 2. According to the fingerprint database established in the first part, the direction information of each color polarizer can be calculated. Therefore, the angle of arrival positioning algorithm can be used to calculate the coordinate information of the camera.

The collection method is as follows: First, a color polarizer is placed in front of a white point light source. We take the position of the color polarizer as the coordinate origin. Then, we place an opaque whiteboard parallel to the color polarizer and turn off other illumination lights except for the white point light source. At this time, a clear color polarization pattern will appear on the whiteboard due to the reflection of the incident light by the whiteboard. The second step is to capture the color polarization pattern appearing on the whiteboard and map the hue value and shooting angle. Here, the distance between the whiteboard and the color polarizer is 5 cm, so the resolution of the color polarization pattern is high.

For white light sources in different positions and directions in the room, only a color polarizer of the same model that does not need to be specially designed is attached because the same color polarizer’s fingerprint is the same. No additional overhead is required. Regarding the portability of the scene, the color polarizer can be plug-and-play. For scenes with multiple light sources, one only needs to know each light source’s relative position and any light source’s absolute position. The interference between the light sources is negligible. Since the color polarizer is close to the light source, the ambient light’s intensity is much lower than the intensity of the light source, so it will not affect the light.

### 5.2. 3D Localization

**Figure 6:**
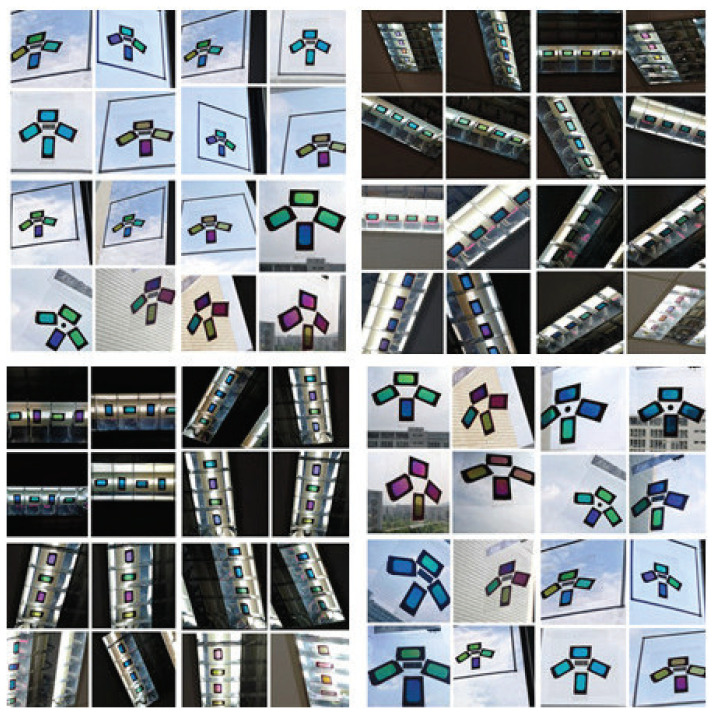
The photo of the color polarizer in different environments.

To achieve coordinate positioning, we first paste several color polarizers at a specific interval on a transparent plane. To ensure the accuracy of the positioning results, we used four-color polarizers S1, S2, S3, and S4, some of the more varied images, as shown in Figure 6, and arranged the four-color polarizers according to a specific relative position. The central positions of S1, S2, S3, and S4 are p1, p2, p3, and p4, respectively. Use the midpoint positions of p1, p2, p3, and p4 as reference points. Move the mobile phone camera to display the color polarization of the three-dimensional area with the color polarizer as the apex as one position of each photo included. For the sampling position r, it derives the hue value h for the chip from the captured photo. The mapping between the direction of the light and the hue value can be constructed by sampling at different locations.

Because the light propagates along a straight line in the same medium, as shown in Figure 3, the color development of the color polarizer at point C is the same as the color observed at points A. Therefore, it can be deduced that if the color polarizer is observed at point C, the color value is the same as the color values of points A and B, and it is evident that the hue value is also the same. Therefore, the fingerprint information can be extended to the entire space.

## 6. Evaluation

We evaluate the performance of the reverse fingerprint collection method from the following aspects:

- The influence of different distances on positioning accuracy;
- The influence of fingerprints collected under different lights and distances;
- The effect of different color polarizer placement on the positioning results;
- The influence of different periods of a day on positioning accuracy;
- The influence of the vertical distance between the light source and camera position on positioning accuracy;
- The influence of different pixel camera on positioning accuracy;
- The influence of different ambient light on positioning accuracy.

### 6.1. Localization Accuracy

**Figure 7:**
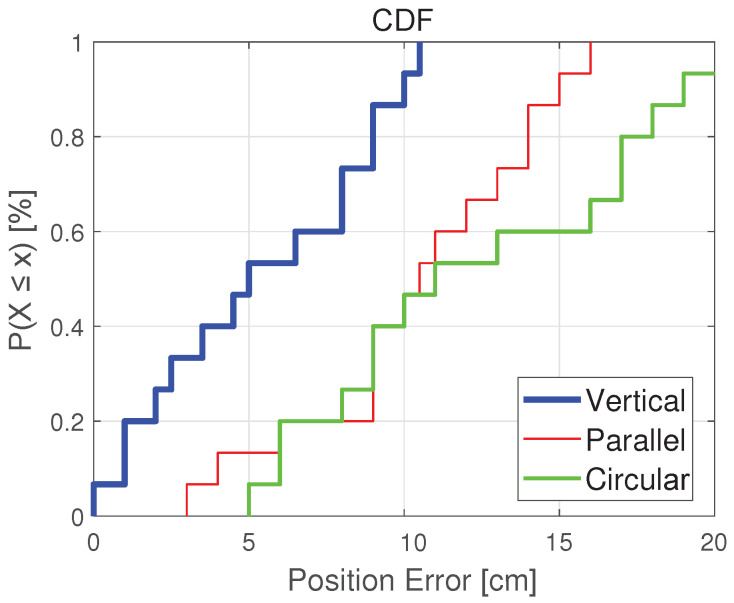
Infuence of different relative-positions on location.

Figure 6 shows the photo of the color polarizer we used to locate. In the experiment, we attached the color polarizer to the light source and moved the phone to different positions in a room of 4 × 4 × 2 m. Figure 7 shows the positioning errors of mobile phones in different positions. It can be seen that the “Vertical” group has the highest positioning accuracy. In 95% of cases, the positioning accuracy can be guaranteed to be less than 10 cm. For the “Parallel” group and the “Circular” group, the positioning accuracy can be guaranteed to be less than 20 cm in 95% of cases. We can see that most positioning errors in the *x* and *y* direction are within 10 cm, with an average error of about 3 cm.

### 6.2. Impact of Different Fingerprints

**Figure 8:**
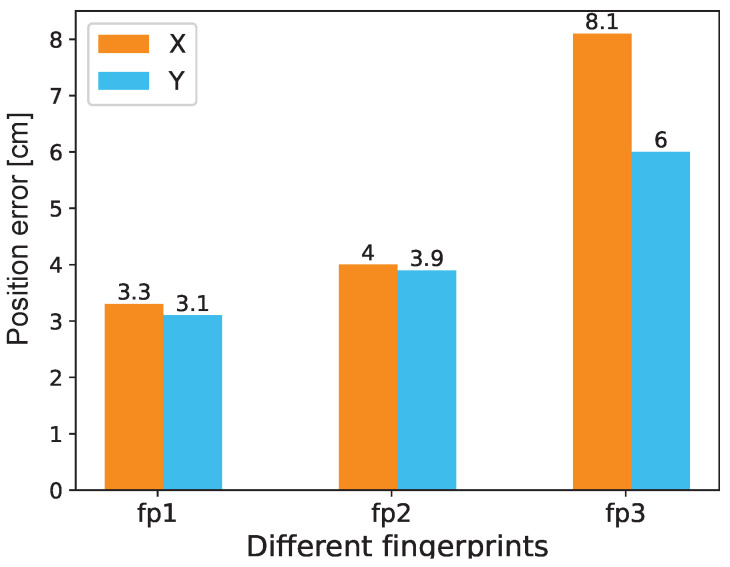
Impact of fingerprints collected in different situations.

We collected fingerprints in different environments to test the impact on the positioning results. (1) In the absence of other ambient light, fingerprints projected by white light, which is called “fp1”, as shown in Figure 8; (2) The fingerprint projected by the white light under the condition of normal ambient light, which is called “fp2”; (3) In the absence of other ambient light, the fingerprint projected by the warm white light, which is called “fp3”. Impact of fingerprints collected in different situations on the results, as shown in Figure 8. It can be observed that in the absence of ambient light, the test results corresponding to the white light group are the best. The test result of the yellow light group without ambient light is the worst. The test results of the white light group with ambient light are only slightly worse than the white light group without ambient light. It can be seen that the reverse fingerprint collection method has high robustness. The influence of ambient light on the accuracy of the fingerprint is small, and the color of the light source has a significant influence on the positioning result.

### 6.3. Impact of Different Color Polarizer Placements

**Figure 9:**
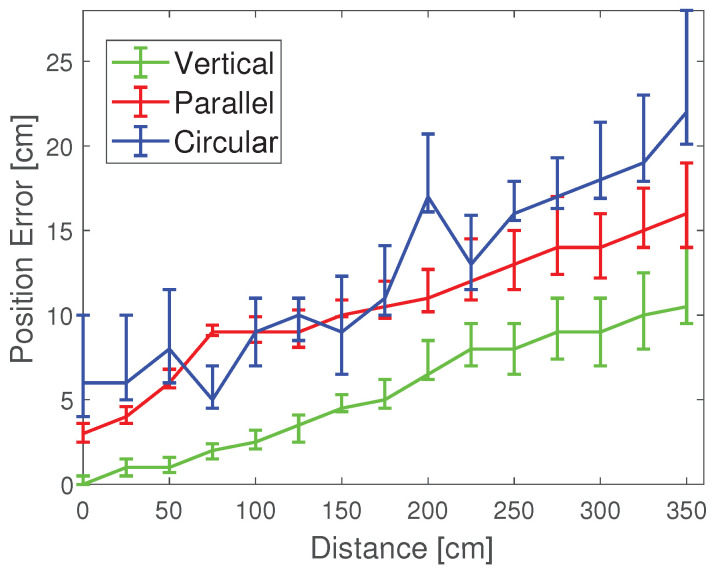
Impact of different color polarizer placements.

The different relative positions of color polarizers also have an impact on the positioning results. We tested three different arrangements of color polarizers, respectively named parallel, vertical, and circular. “Parallel” means that the four color-polarizers have only translated, not rotated; “Vertical” means that the four-color polarizers rotate by 90 degrees each based on translation; “Circular” means that four color-polarizers are rotated 60 or 120 degrees while being translated. It can be seen that the test result of the parallel group is the worst, and the test result of the vertical group is the best. As shown in Figure 9, the position error of the circular group is between the vertical and the parallel group.

### 6.4. Influence of Different Time Periods of a Day

**Figure 10:**
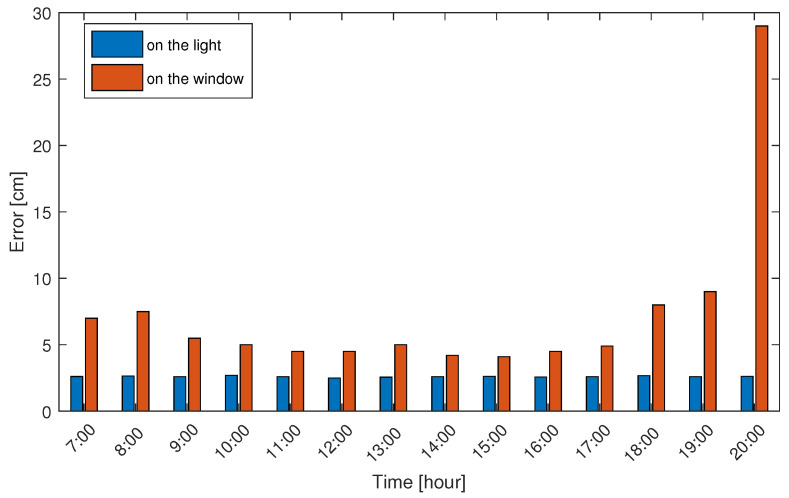
The influence of different time periods of a day on positioning accuracy.

We have observed that there are apparent changes in light in the early morning and noon, and there are also differences in outdoor light on rainy and sunny days. In order to explore the influence of different periods of the day on positioning, we used the same camera and shooting angle to collect photos of the color polarizer during the period from 7:00 to 20:00 during the day. Figure 10 shown the difference in positioning accuracy overtime when the color polarizer is attached to the ceiling light and the window. We found that the color polarizers attached to the windows changed significantly with different times of the day. The color polarizers on the window use sunlight as the light source, so the positioning error in the evening will increase with the change of light color and intensity, as shown in Figure 10. When the color polarizer attached to the ceiling lamp is positioned, it is not affected by different periods of the day.

### 6.5. Influence of Vertical Distance between Light Source and Camera Position

**Figure 11:**
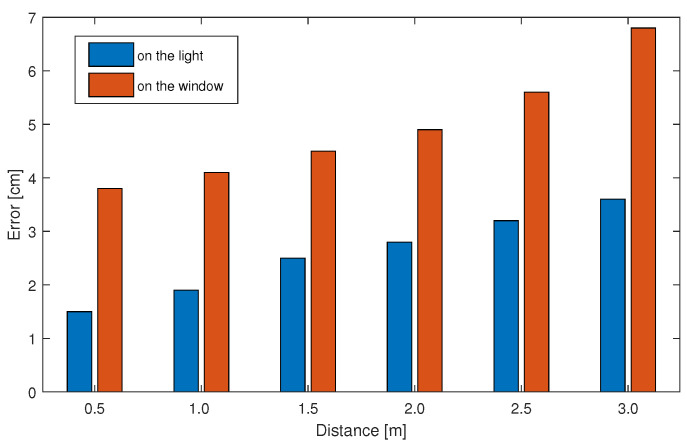
Influence of vertical distance between light source and camera position.

To study the difference in positioning accuracy between the color polarizer and the camera at different vertical distances. We start with a vertical distance of 0.5 m between the color polarizer and the camera and measure points that are on a line perpendicular to the ground. The positioning accuracy is measured every time the vertical distance is increased by 0.5 m. We found that positioning accuracy gradually improved as the distance decreased. When the distance between the camera and the color polarizer is only 0.5 m, the positioning error can be reduced to within 2 cm under the lamp and within 4 cm under natural light. As shown in Figure 11, when the vertical distance between the camera and the color polarizer is 3 m, the positioning accuracy under the lamp is 4–5 cm on average.

### 6.6. Impact of Different Pixel Camera

**Figure 12:**
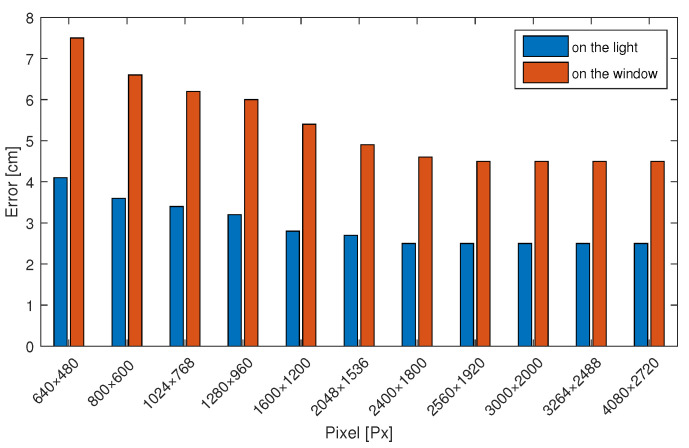
Influence of different pixel camera on positioning accuracy.

In order to study the impact of cameras with different pixels on the positioning accuracy, we compared the differences in positioning accuracy caused by photos from 600 × 480 pixels to 4080 × 2720 pixels. We found that when the camera pixel is only 30W because the color polarizer itself is small, a color polarizer occupies a small photo area. Excluding the pixels of the outermost circle of the color polarizer (because it is uncertain that the outermost pixels belong to the imaging range of the color polarizer), the number of remaining pixels is small, so the error obtained is larger. As shown in Figure 12, the positioning accuracy obtained by a 4080 × 2720 pixel camera is 2–4 cm greater.

### 6.7. Impact of Different Ambient Light

There are some differences in the positioning results of color polarizer under different environments. As shown in Table 1, the group with no ambient light interference has the best positioning effect, because the test environment in this case is the same as the fingerprint collection environment. In the natural light environment (the color polarizer is directly placed on the window and positioned with sunlight), the positioning results are the worst. We consider that the main reason for the high positioning error is the complex environment outside the window. Both the ground and the sky have colors, and it is difficult to distinguish whether the collected colors are generated by the color polarizer or the background refraction. When other lights are on at the same time, the positioning accuracy is not affected much. Therefore, the fingerprint locating method proposed in this paper has better environmental robustness.

## 7. Conclusions

This paper improves the fingerprint position method and implements the reverse fingerprint construction method. The advantages of using the reverse fingerprint collection method are as follows: First, it does not need to take multiple photos to build a fingerprint dataset and take only one picture to build the fingerprint library of the whole space. Second, the fingerprints collected in one environment can be reused in multiple different environments. Third, it avoids the decrease in positioning accuracy because of insufficient sampling density. We theoretically proved that the acquisition of fingerprint information could be used for multiple conditions. Then, we compared the effect of different relative positions of the color polarizer on the positioning accuracy. The evaluation results show that reverse fingerprint position method achieves an average localization error of about 3 cm in 2D, and in 95% of cases, the positioning accuracy can be guaranteed to be less than 10 cm. This paper has high practical significance at the application level for the indoor positioning of intelligent cars, UAVs, and other equipment. Taken together, the findings of this study have a number of important implications for future practice.

This limitation of this study is that the positioning process requires shooting towards the light source, so it is more suitable for smart cars or drones with cameras. Further research might explore that integrate the part that recognizes the LED-ID into the positioning algorithm.

## Figures and Tables

**Table 1:** Different color polarizer relative position.

Environment	Photo	Mean Error
Natural Light	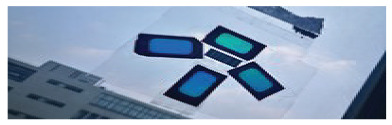	25.45 cm
W/ Luminaires	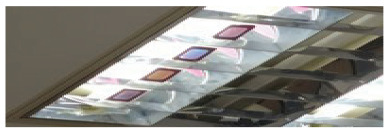	7.79 cm
W/O Luminaires	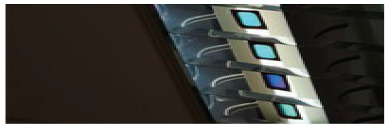	3.3 cm
